# The Combination of *Bacillus Thuringiensis* and Its Engineered Strain Expressing dsRNA Increases the Toxicity against *Plutella Xylostella*

**DOI:** 10.3390/ijms23010444

**Published:** 2021-12-31

**Authors:** Ying-Xia Jiang, Jin-Zhi Chen, Miao-Wen Li, Ben-Hu Zha, Peng-Rong Huang, Xue-Mei Chu, Jing Chen, Guang Yang

**Affiliations:** 1 State Key Laboratory of Ecological Pest Control for Fujian and Taiwan Crops, Institute of Applied Ecology, Fujian Agriculture and Forestry University, Fuzhou 350002, China; 18270821925@163.com (Y.-X.J.); chjinzhi@yeah.net (J.-Z.C.); miaowenLee@163.com (M.-W.L.); benhu_zha@163.com (B.-H.Z.); pengronghuang@163.com (P.-R.H.); 15165546235@163.com (X.-M.C.); jingchen0816@163.com (J.C.); 2 Joint International Research Laboratory of Ecological Pest Control, Ministry of Education, Fuzhou 350002, China; 3 Key Laboratory of Integrated Pest Management for Fujian-Taiwan Crops, Ministry of Agriculture and Rural Affairs, Fuzhou 350002, China; 4 Key Laboratory of Green Control of Insect Pests, Fujian Province University, Fuzhou 350002, China; 5 Ministerial and Provincial Joint Innovation Centre for Safety Production of Cross-Strait Crops, Fujian Agriculture and Forestry University, Fuzhou 350002, China

**Keywords:** *Plutella xylostella*, entomopathogen, RNA interference, pest management

## Abstract

RNA interference (RNAi) has been developed and used as an emerging strategy for pest management. Here, an entomopathogen *Bacillus thuringiensis* (*Bt*) was used to express the dsRNA for the control of *Plutella xylostella*. A vector containing a 325-bp fragment of the conserved region of *P. xylostella* arginine kinase gene (*PxAK*) flanking in two ends with the promoter Pro3α was developed and transferred into *Bt* 8010 and BMB171, and consequently engineered *Bt* strains 8010AKi and BMB171AKi expressing dsRNA of *PxAK* were developed. The two engineered *Bt* strains were separately mixed with *Bt* 8010 in a series of ratios, and then fed to the *P. xylostella* larvae. We found that 8010:8010AKi of 9:1 and 8010:BMB171AKi of 7:3 caused a higher mortality than *Bt* 8010. *PxAK* expression levels in the individuals treated with the mixtures, 8010AKi and BMB171Aki, were lower than that in the control. The intrinsic rate of increase (*r*) and net reproductive rate (*R*_0_) of the population treated with 8010:8010AKi of 9:1 were lower than those of the population treated with *Bt* 8010 or 8010AKi. We developed a *Bt*-mediated insect RNAi for the control of *P. xylostella* and demonstrated a practical approach to integrating the entomopathogen with RNAi technique for the pest management.

## 1. Introduction

RNA interference (RNAi) is a potential genetic tool for pest management [1,2,3]. In previous studies, it has been proven that transgenic plants/microorganism expressing double-strand RNA (dsRNA) of insect pest gene(s) can disturb the insect growth and development, and even lead to death [4,5]. The larval stunting and mortality of western corn rootworm *Diabrotica virgifera virgifera* LeConte (Coleoptera: Chrysomelidae) and reduction in plant damage are observed after *D. virgifera virgifera* feeds on transgenic corn expressing dsRNA of V-ATPase A gene (*V2AYP*) [6]. The same phenomena are reported in dsRNA-expressed transgenic plants, such as *Solanum tuberosum* Linnaeus [5], *Nicotiana tobacum* Linnaeus and *Gossypium hirsutum* Linnaeus cv. Xu-142 [7], and *Arabidopsis thaliana* (Linnaeus) Heynhold [8,9].

Another efficient way of using RNAi technology to control insect pests is through the engineered bacteria expressing dsRNA. Among these engineered bacteria, the *Escherichia coli* (Migula) Castellani and Chalmers strain HT115, which is deficient in RNase III enzyme, is used to produce dsRNA [10,11]. RNase III widely exists in bacteria and can process long dsRNA into siRNA [12,13]. In order to obtain long dsRNA, the defective strain of RNase III is chosen in the RNAi studies [14,15]. Reports state that the survival rates of *Spodoptera exigua* Hübner (Lepidoptera: Noctuidae)feeding on *E. coli* HT115 expressing dsRNA of chitin synthase gene A (*SeCHSA*) is lower than that of individuals feeding on controls [10]. When HT115 expressing dsRNA of integrin β_1_ subunit gene was coupled with *E. coli* expressing Cry1Ca or a commercial *Bacillus thuringiensis* Berliner (*Bt*), a high insecticidal efficacy was achieved [11]. Interestingly, with the help of p19, a siRNA-binding protein, both wild type *E. coli* and HT115 with RNase III expression restored can accumulate a mass of siRNAs to achieve an efficient interference [16].

The diamondback moth, *Plutella xylostella* Linnaeus (Lepidoptera: Plutellidae), is one of the most destructive pests in cruciferous crops and causes annual economic losses of up to $ 4–5 billion in the world [17] and $0.77 billion in China [18]. *Bt* is commonly used for the biological control of *P. xylostella*. However, *P. xylostella* has developed resistance to 97 kinds of pesticides including *Bt* [19], which makes it difficult to control. Therefore, more effective management strategies are required for the control of *P. xylostella*.

In this study, *P. xylostella* arginine kinase (*PxAK*) was chosen as the target gene of RNAi as it only exists in invertebrates and some protozoa, and conducts energy metabolism in a different way compared to mammals [20,21]. Furthermore, the high mortality of *P. xylostella* was gained by feeding larvae with the synthesized *PxAK* dsRNA or *E. coli* HT115 expressing *PxAK* dsRNA in our lab (unpublished data). To achieve a new approach for *P. xylostella* management, entomopathogen and RNAi technique were integrated into our research. *Bt* 8010, a commercial *Bt* strain and BMB171, a *Bt* mutant without Cry toxins, were chosen as the entomopathogen for the study. An RNAi vector for *PxAK* was constructed and transformed into *Bt* 8010 and BMB171 to obtain engineered *Bt* 8010AKi and BMB171AKi expressing dsRNA of *PxAK*. The two engineered *Bt* strains were separately mixed with *Bt* 8010 in different ratios and fed to *P. xylostella* larvae to screen the mixture with a better control of *P. xylostella* than *Bt* through the toxicity bioassay and the age-stage, two-sex life table analysis.

## 2. Results

### 2.1. Engineered Bt Expressing dsRNA of PxAK

**Figure 1:**
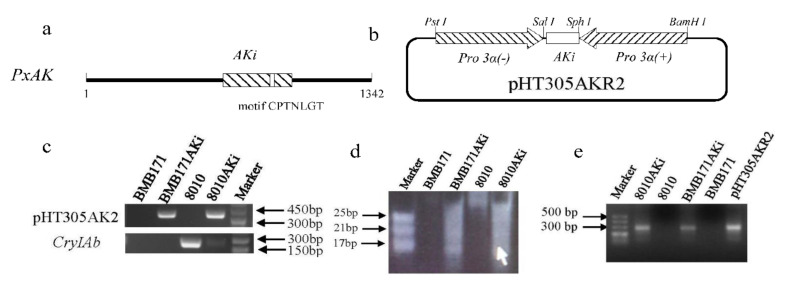
Construction of a recombinant *Bt* expressing dsRNA of *PxAK*. (**a**) The schematic representation of *PxAK* and the AKi fragment used in the experiment. The white box stands for the motif CPTNLGT. (**b**) A recombinant *Bt* pHT305AK2 plasmid containing the expression cassette for dsRNA of AKi; Pro3α(−) on the left of AKi; Pro3α(+) on the right of AKi. (**c**) Verification of engineered *Bt* strains by PCR to check the presence of vector pHT305AK2 and Cry gene. The PCR product for vector and Cry gene were 396 bp and 238 bp, respectively. (**d**) Detection of small RNA in engineered *Bt* strains by 19% PAGE-Urea gel. (**e**) Detection of the expression of *AKi* in engineered *Bt* strains.

A 325-bp PCR product of *PxAK* fragment (AKi) (Figure 1a and Figure S1a) was inserted between two pro3α promoters (Figure 1b and Figure S1a) to obtain the recombinant vector pHT305AKR, and the *Sph*I site in pHT304 was mutated to get the pHT305AKR2 plasmid (Figure S1b). The pHT305AKR2 plasmid was transformed into *Bt* 8010 and BMB171 to produce engineered bacteria 8010AKi and BMB171AKi (Figure S1c). The transformed 8010AKi and BMB171AKi were verified by the presence of pHT305AKR2 plasmid with the PCR product of 396 bp and the presence of Cry gene in 8010AKi was also verified by the PCR product of 238 bp (Figure 1c). The stability of the pHT305AKR2 plasmid was >90% in 30 generations when 8010AKi was cultured in LB media containing erythromycin (Figure S2). No long dsRNA of AKi was detected in the engineered strain, however a smeared region ranging from 17 bp to 25 bp was observed in the RNA extracted from the engineered *Bt* (Figure 1d) and 304 bp of AKi fragment was PCR amplified from the cDNA of the engineered *Bt* (Figure 1e).

### 2.2. Toxicity of Engineered Bt against P. xylostella

**Figure 2:**
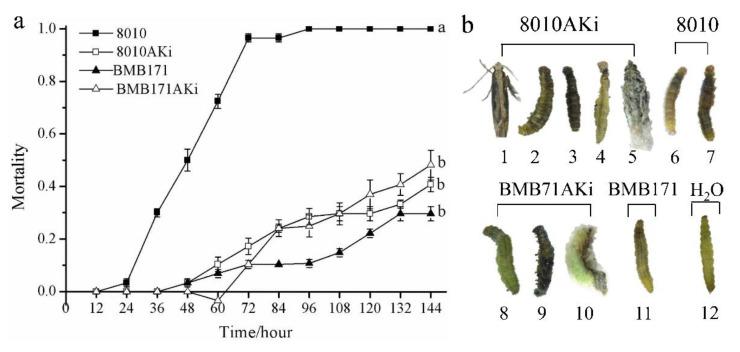
Insecticidal activity of engineered *Bt* strains against *P. xylostella.* (**a**) Oral toxicity of bacteria against *P. xylostella*. Each group contained 30 4th-instar larvae and mortality was measured every 12 h. (**b**) Effect of bacteria on *P. xylostella* development. In 1, the right antenna was shorter than the left; in 2, and 8, body appeared to exhibit shrinkage; in 3 and 9, the whole body turned blackish or green and the thorax appeared to exhibit shrinkage; in 4, thorax appeared to exhibit extreme shrinkage; in 5, the adult failed to break the cocoon; in 10, the pupa failed to ecdysis; in 6 and 7, the body turned black and erosive; in 11, the body was elongated; in 12, no abnormal phenotype was observed.

The larvae started to die on the 2nd day after feeding on the cabbage leaves treated with the 8010AKi cultures, and most larvae died in the next 12 h (Figure 2a). The mortality of larvae treated by 8010AKi reached 46.7% on the 6th day, which caused less mortality of *P. xylostella* than *Bt* 8010; the larvae treated with BMB171AKi began to die on the 3rd day, and the mortality reached up to 53.3% on the 6th day, which was higher than that treated with BMB171 (*F* = 44.789, *df*_1_ = 3, *df*_2_ = 8, *p* = 0.000, Figure 2a). For daily cumulative mortality, 8010 had a higher effect on *P. xylostella* than 8010AKi (*t* = 6.770, *df* = 11, *p* = 0.000), while BMB171AKi higher than BMB171 (*t* = −2.216, *df* = 11, *p* = 0.049).

For 8010AKi treated individuals, the head, thorax and even the whole body of dead larvae turned blackish, which was similar to the phenotype of dead larvae caused by *Bt* 8010, and the thorax and abdominal regions appeared to exhibit the typical shrinkage caused by the suppression of *PxAK* (Figure 2b). The dead larvae caused by BMB171AKi showed a similar phenotype with the dead larvae caused by 8010AKi (Figure 2b).

### 2.3. Toxicity of the Mixtures of Bt 8010 and Engineered Bt against P. Xylostella

**Figure 3:**
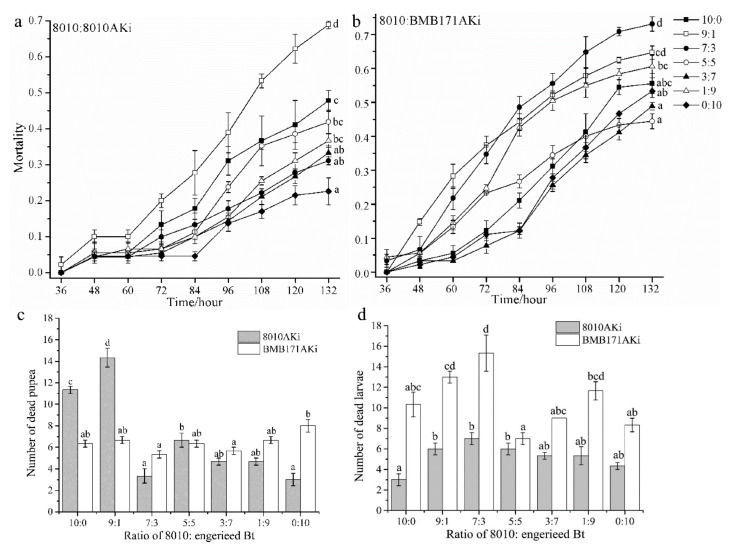
Mortality of *P. xylostella* caused by the mixtures of *Bt* 8010 and engineered *Bt* expressing dsRNA of *PxAK*. The mortality of *P. xylostella* caused by the bacteria mixtures in different ratios were collected every 24 h and three biological replications were performed for each treatment. (**a**) *Bt* 8010:8010AKi; (**b**) *Bt* 8010:BMB171AKi. The total number of dead *P. xylostella* caused by the mixtures of *Bt* 8010 and engineered *Bt* in different ratios were recorded and three biological replications were performed for each treatment. (**c**) Pupae; (**d**) Larvae. The bars represent the mean ±  SE of each treatment. Different lowercase letters represent significant differences among the mixtures of different ratios for 8010:8010AKi and 8010:BMB171AKi separately by Tukey test in one-way ANOVA (*p* < 0.05).

There were significant differences in the mortality of *P. xylostella* at 132 h after treatment among mixtures of different ratios (*F* = 36.187, *df*_1_ = 6, *df*_2_ = 14, *p* = 0.000 for 8010AKi group; *F* = 17.390, *df*_1_ = 6, *df*_2_ = 14, *p* = 0.000 for BMB171AKi group), and 8010:8010AKi of 9:1 and 8010:BMB171AKi of 7:3 caused the highest mortalities of *P. xylostella* with the values of 68.9% and 73.1%, which was about 20% higher than that of the *Bt* 8010 (Figure 3a,b). The pupal mortality caused by 8010:8010AKi of 9:1 was higher than mixtures of other ratios (*F* = 56.429, *df*_1_ = 6, *df*_2_ = 14, *p* = 0.000; Figure 3c). The larval mortality caused by 8010:BMB171AKi was higher than that by 8010:8010AKi (*t* = −7.980, *df* = 20, *p* = 0.000) and 8010:BMB171AKi of 7:3 caused a higher larval mortality than that by *Bt* 8010 or BMB171AKi (*F* = 9.052, *df*_1_ = 6, *df*_2_ = 14, *p* = 0.000; Figure 3d).

**Figure 4:**
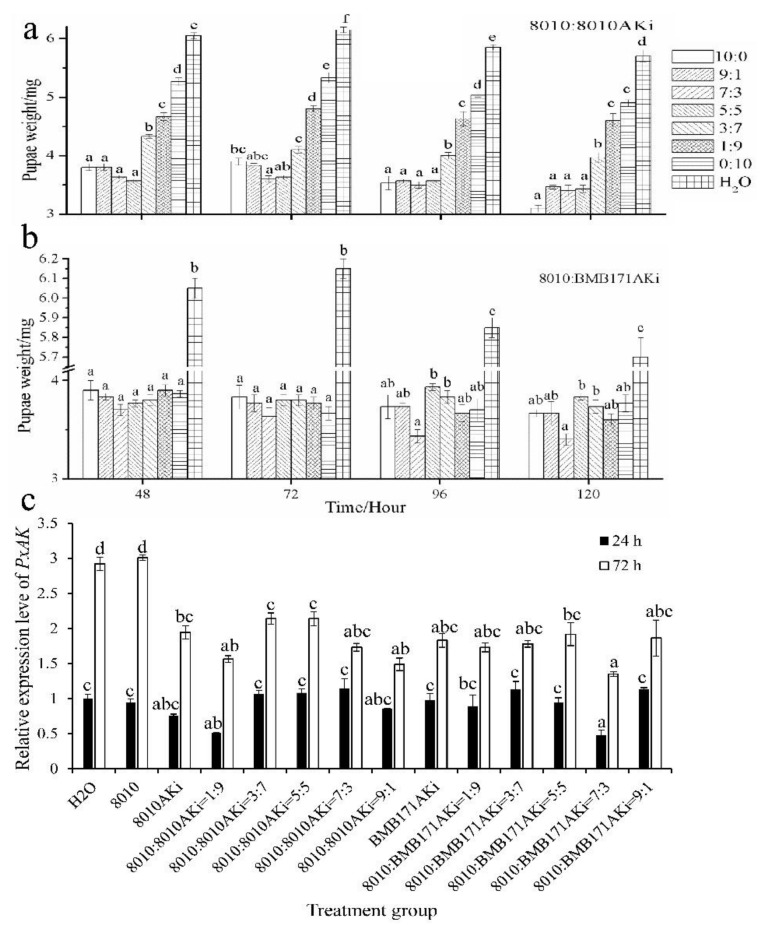
Effects of the mixtures of 8010 and engineered *Bt* on *P. xylostella* pupal weight and *PxAK* expression. The pupal weight of *P. xylostella* treated with the bacteria mixtures of different ratios. (**a**) 8010:8010AKi; (**b**) 8010:BMB171AKi. Values represent the mean ± SE of pupal weight of *P. xylostella* from three biologically independent replicates of each treatment at each time point. (**c**) *PxAK* expression in *P. xylostella* treated with 8010:8010AKi or 8010:BMB171AKi of different ratios. *PxRPL8* was used as the reference gene, and the *PxAK* expression in *P. xylostella* treated with H_2_O was the control. The bars represent the mean ± SE of relative expression of *PxAK* mRNA transcripts from three biologically independent replicates of each treatment. The different lowercase letters represent significant differences among the mixtures of different ratios at each time point by Tukey test in one-way ANOVA (*p* < 0.05).

When compared with the individuals treated with water, the weight of pupae treated by the mixtures was decreased significantly (*F* = 301.469, *df*_1_ = 7, *df*_2_ = 16, *p* = 0.000 for 8010:8010AKi; *F* = 121.808, *df*_1_ = 7, *df*_2_ = 16, *p* = 0.000 for 8010:BMB171AKi); 8010:8010AKi had a greater impact on pupae weight than 8010:BMB171AKi (*t* = 2.905, *df* = 23, *p* = 0.008) (Figure 4a, b). The expression of *PxAK* in individuals treated by 8010:8010AKi, 8010:BMB171AKi, 8010AKi and BMB171AKi, respectively, was lower than that in individuals treated by *Bt* 8010 and water at 72 h (*F* = 20.639, *df*_1_ = 13, *df*_2_ = 28, *p* = 0.000, Figure 4c).

### 2.4. Growth and Development Characteristics of P. xylostella Feeding on the Mixtures of Bt and Engineered Bt

Compared to the individuals treated with *Bt* 8010, the durations of larvae and preadults were prolonged among all the mixtures. Despite the pre-adults durations of individuals treated with 8010:8010AKi of 3:7, the durations of larvae and pre-adults among all the mixtures were not different from the individuals treated with 8010AKi. Although there were no significant differences on the durations of adults and male adults, the duration of female adults in the individuals treated with 8010:8010AKi of 3:7, 5:5 and 9:1 was shorter than individuals treated with *Bt* 8010. (Table S1).

There was no significant difference on larval mortality between individuals treated with the mixtures and individuals treated with *Bt* 8010 or 8010AKi, as well as the fecundity and oviposition period although the total preoviposition period of individuals treated with the mixtures were prolonged compared to that of individuals treated with *Bt* 8010. The lifespan and the pre-adult survival rates of individuals treated with 8010:8010AKi of 9:1 were significantly lower than the individuals treated with *Bt* 8010, 8010AKi or H_2_O. Totally, 8010:8010AKi of 9:1 caused significant difference in most population parameters compared with *Bt* 8010 among all the mixtures. (Table S1).

### 2.5. Effects of the Mixtures of Bt and Engineered Bt on P. xylostella Population Dynamics Parameters

The intrinsic increase rate (*r*) and finite increase rate (*λ*) of the individuals treated with the mixtures were significantly lower than those of the individuals treated with *Bt* 8010 or 8010AKi, except the individuals treated with 8010:8010AKi of 5:5; *r*, *λ* and the net reproductive rate (*R*_0_) of the individuals treated with 8010:8010AKi of 9:1 was significantly lower than those of the individuals treated with *Bt* 8010, 8010AKi or H_2_O; the mean generation period (*T*) of the individuals treated with mixtures was longer than that of the individuals treated with *Bt* 8010 (Table 1).

In general, compared with *Bt* 8010, 8010:8010AKi of 9:1 could delay the development of *P. xylostella*, lower the survival rate in the precocious stage, shorten the duration of female moths, decrease the lifespan of adults and female moths (Table S1), and then suppress *r* and *λ* of the population (Table 1).

## 3. Discussion

In our experiment, it was more difficult to transform the exogenous plasmid into *Bt* 8010 than BMB171. The transformation efficiency of wild-type strain is lower than that of plasmid-free or less plasmid strains, which may be due to the plasmid incompatibility [22]. The diversity and abundance of endogenous plasmids are the main factors affecting the frequency of transferring the foreign plasmid into the strain and the stability of the transformed strain [22,23,24]. Therefore, the low transformation efficiency of *Bt* 8010 in our study might be due to the fact that *Bt* 8010 has endogenous plasmids. The other factor affecting the transformation efficiency is the nature of plasmid. The same plasmid has different transformation efficiency in different strains [25]. The plasmid pHT304 used in this study is a low-copy-number plasmid [26], which also might lead to a low transformation efficiency.

In 8010AKi and BMB171AKi, siRNAs of 17–25 bp, rather than long dsRNA, was accumulated to inhibit the expression of *PxAK* in *P. xylostella* through feeding. Since *Bt* 8010 and BMB171 are RNase III non-defective strains, the long dsRNA might be cut into siRNA by RNase III. In *Drosophila* S2 cells, siRNA produced by *E. coli* RNase III is more potent than dsRNA [13]. Also, GFP fluorescence intensity in *Caenorhabditis elegans* (Maupas) Dougherty is reduced by non-RNase III-defective *E. coli* strain expressing GFP dsRNA [12]. Both pieces of research provide evidence that siRNA produced by RNase III can lead to effective RNAi. For further research, RNase III-knockout *Bt* 8010AKi needs to be developed to examine whether RNase III-defective bacteria can cause a high RNAi efficacy in *P. xylostella*.

AK is commonly known as an energy metabolizer for muscle contraction [20]. AK is also involved in ecdysone regulation in *Drosophila* [27] and adult fertility and stress response in *Tribolium castaneum* Herbst (Coleoptera: Tenebrionidae) [28]. We observed that the thorax and abdominal regions of *P. xylostella* larvae appeared to exhibit typical shrinkage, some larvae failed to exuviate and some pupae could not break out after 8010AKi or BMB171AKi treatment, which was probably caused by the disorder of energy metabolism for muscle development after *PxAK* was inhibited. Similarly, ingestion of transgenic plants expressing dsRNA of *AK* significantly retards the larval growth and development and even causes the death in *Helicoverpa armigera* Hübner (Lepidoptera: Noctuidae) [8] and *P. xylostella* [9]. Generally, insect pests die with a brown or black body after *Bt* infection [29], which was also observed in dead *P. xylostella* larvae after 8010AKi treatment. Therefore, 8010AKi worked on *P. xylostella* through not only its original toxicity but also RNAi of *PxAK*.

Although 8010AKi was less effective than the *Bt* 8010, the mixture of 8010:8010AKi of 9:1 and 8010:BMB171AKi of 7:3 caused higher mortality than *Bt* 8010. Engineered bacteria expressing dsRNA have been mixed with *Bt* for a better toxicity against insect pests. For example, *Bt* mixed with *E. coli* expressing dsRNA of integrin β_1_ subunit gene (*dsINT*) can cause more death of *S. exigua* [11]. Similar pieces of research have been conducted regarding *Spodoptera littoralis* Boisduval (Lepidoptera, Noctuidae) [30] and *Maruca vitrata* Fabricius (Lepidoptera: Crambidae) [31] for a remarkable enhancement of toxicity. The mixtures of 8010:8010AKi showed that 8010:8010AKi of 9:1 had higher toxicity than *Bt* 8010, and life table analyses showed that 8010:8010AKi of 9:1 decreased the survival rate, delayed the development, and suppressed the population compared with *Bt* 8010, indicating that the mixture could have a better control of *P. xylostella*.

## 4. Materials and Methods

### 4.1. Insects and Bacteria

Two strains of *P. xylostella* were used in this study. The insecticide-susceptible strain (Fuzhou-S) was reared on radish, *Raphanus sativus* Linnaeus seedlings [32], and the artificial diet strain obtained from the Institute of Zoology, Chinese Academy of Science (Beijing), was reared on the artificial diet [33]. The pupae were collected in the bottle with a cotton wool containing 10% honey solution as extra-nutrition for adults, and eggs were collected by a plate cover card stained with vegetable powder hung in the cup. Insects were reared in a controlled laboratory condition (25 ± 1 °C, 65% ± 5% RH and 16 h:8 h of L:D) in the growth chambers. *Bt* 8010 and BMB171 was cultured in LB media at 30 °C. Both strains had no resistance to any antibiotics.

### 4.2. Construction of RNAi vector of PxAK

Primers 01F/01R and 02F/02R (Table S2) were used to amplify the sequence of pro3α in the plasmid pHT305a [26] and the amplified sequences were named as pro3α(+) and pro3α(−) separately. PCR reaction was carried out using the PrimeSTAR^®^ HS DNA Polymerase ((TaKaRa, Shiga, Japan) with the following conditions: one cycle of 98 °C for 3 min; 20 cycles of 98 °C for 10 s, 60 °C for 30 s with 0.5 °C reduced per cycle, and 72 °C for 90 s; 25 cycles of 98 °C for 10 s, 50 °C for 30 s, 72 °C for 90 s; and the final cycle of 72 °C for 5 min. The 325-bp fragment of *PxAK* (GenBank: HQ327310.1) (AKi), containing CPTNLGT motif sequence, was chosen as the target of RNAi. AKi was cloned from the cDNA of *P. xylostella* by primers 03F/03R (Table S1) with the same reaction procedure except for the extension time changed from 90 s to 45 s, then connected to plasmid PUM-T (BioTeke, Beijing, China) to obtain plasmid PUMAK.

The plasmid PUMAK was digested with *Sph*I (TaKaRa) to obtain the AKi fragment. The AKi was linked with pro3α(+) to produce the fragment of AKi+pro3α(+). The plasmid pHT305a was digested with *Bam*HI (TaKaRa) to obtain the fragment of pHT304 [26]. The fragment AKi+pro3α(+) was linked with the fragment of pHT304 to obtain the intermediate cloning vector pHT304+pro3α(+)+AKi. At the final step, the pro3α(−) was inserted into the intermediate vector to get the plasmid pHT305AKR containing pHT304+pro3α(+)+AKi+pro3α(−) (Figure S3). Slicing by the Overlapping Extension Polymerase Chain Reaction and double enzyme digestion were performed to remove *Sph*I restriction site from pHT304 to obtain the final plasmid pHT305AKR2 (See additional methodology). The newly constructed plasmid was verified through *Sph*I digestion.

### 4.3. Construction of Engineered Bt

According to Dower’s and Masson’s methods [34,35], the competent cells of *Bt* 8010 and BMB171 were collected at the OD_600_ of 1.2–1.5 and washed with sterile water, and then electro-transformed with plasmid pHT305AKR2 at 2.5 kv using the MicroPulser (Bio-Rad, CA, USA). The engineered bacteria 8010AKi and BMB171AKi were confirmed through PCR with the primers Bt-F/Bt-R to examine the presence of the pHT305AKR2, and with the primers Cry1F/Cry1R to examine the presence of *Cry* gene [29] (Table S2). The plasmid stability in 8010AKi was detected by culturing the bacteria in LB medium with or without erythromycin according to the method described by Harwood and Cutting [36]. The total RNA was extracted by using the TRIzol^®^ reagent (Ambion, California, USA) and examined by 1% agarose gel. Small RNA was extracted by the E.Z.N.A.T. miRNA kit (OMEGA, Doraville, USA) and examined by 19% PAGE-Urea gel according to Huang’s description [16]. The transcription of AKi in engineered bacteria was also detected through PCR with their cDNA and specific primer pair AKiF/AKiR (Table S2). PCR reaction was carried out using 2× Taq Master Mix (Vazyme, Nanjing, China) with the following conditions: one cycle of 95 °C for 5 min; 35 cycles of 95 °C for 30 s, 55 °C for 30 s and 72 °C for 30 s; and the final cycle of 72 °C for 5 min.

### 4.4. Feeding Bioassay

The bacterial solutions (8010AKi, BMB171AKi, 8010 and BMB171) were prepared after overnight culture and diluted to 1 OD_600_. Then cabbage leaves were immersed in the diluted bacterial solution for 2 h and then transferred into petri dishes (90-mm diameter) after being air dried for feeding bioassay. Thirty 3^rd^-instar larvae were transferred to the leaves in the petri dish, and then covered with a cup. Three biological replications were performed for each treatment. All the treated larvae were reared in the same condition as previously described. The leaves were replaced with the same newly treated ones every 24 h and the number of dead individuals was recorded every 12 h for 6 d. The mortality of *P. xylostella* at different times was carried out by the formula of Schneider Orelli [37], the group treated by H_2_O was the control. One-way ANOVA was carried out to analyze the differences in the mortality of *P. xylostella* among the treatments followed by Tukey test (*p* < 0.05) and paired *t* test was used to analyze the differences in the daily cumulative toxicity against *P. xylostella* between *Bt* and its engineered bacteria (*p* < 0.05) (SPSS 21.0, SPSS Inc. Chicago, IL, USA).

For mixture bioassay, 8010 was mixed with 8010AKi or BMB171AKi at the following ratios: 10:0, 1:9, 3:7, 5:5, 7:3, 9:1, 0:10. The artificial diet [33] was soaked in the mixture and then air-dried to feed *P. xylostella*. All the treated larvae were reared in the same condition as previously described. The diets were replaced every 24 h and the number and phenotype of dead individuals were examined every 12 h, and the weight of pupae every 24 h. Thirty 3^rd^-instar larvae were used in each treatment and three biological replications were performed for each treatment. One-way ANOVA was carried out to analyze the differences in the mortality of *P. xylostella* at different times, the number of dead *P. xylostella* larvae or pupae and pupal weight caused by the mixtures of 8010:8010AKi or 8010:BMB171AKi in different ratios, followed by Tukey test (*p* < 0.05) and paired *t* test was used to analyze the differences in the larval mortality caused by mixture groups of 8010:8010AKi and 8010:BMB171AKi (SPSS 21.0, SPSS Inc. Chicago, IL, USA).

### 4.5. Quantification of PxAK Gene Expression

For each treatment, five individuals of *P. xylostella* were collected after feeding on diet for 72 h and three biological replications were performed. The total RNA of *P. xylostella* was extracted using the TRIzol reagent (Trans, Beijing, China). The first-strand cDNA was synthesized by the GoScript ^®^ Kit (Promega, Madiso, WI, USA). The qPCR was carried out using the GoTaq^®^ qPCR Kit (Promega, Madiso, WI, USA) with *PxAK*-specific primers AKF/AKR and primers RF/RR for the reference gene, *P. xylostella* ribosomal protein gene *PxRPL8* (GenBank: NM_001305488) (Table S2) at the conditions: 95 °C for 3 min, followed by 40 cycles of 95 °C for 15 s and 60 °C for 30 s. The 2^−ΔΔCT^ method was used to calculate the relative gene expression. One-way ANOVA was carried out to analyze *PxAK* expression caused by the mixtures of 8010:8010AKi and 8010:BMB171AKi in different ratios, followed by Tukey test (*p* < 0.05) (SPSS 21.0, SPSS Inc. Chicago, IL, USA).

### 4.6. Life Table Analysis of Laboratory Population

For life table studies, every egg of *P. xylostella* was transferred into a disposable sauce cup of 25 mL containing the artificial diet treated with the same method mentioned in the mixture bioassay. The diets were replaced every 24 h. The developmental stage of *P. xylostella* was recorded every day and the number of newly-produced eggs every other day.

The raw data were recorded according to the age-stage two-sex life table theory [38] to construct two-sex life tables of the population of *P. xylostella* in the laboratory. The essential parameters of the life tables were computed by TWOSEX-MSChart (20180810-B200000, version: 2018.8.10) [39]. The mean values and standard errors of population dynamics parameters were calculated using the bootstrap method. The number of repetitions of bootstrap was 200000, and the differences between treatments were tested by the paired bootstrap test.

## 5. Conclusions

In summary, this is the first time dsRNA has been expressed in the entomopathogenic bacterium, *Bt*. We found that the mixture of *Bt* and engineered *Bt* expressing dsRNA could have a synergistic effect for the control of *P. xylostella,* providing a novel approach to integrating entomopathogen with the RNAi technique for the control of insect pests.

## Figures and Tables

**Table 1:** Population dynamics parameters of *P. xylostella* in different treatments (Mean ± SE).

Treatment	Intrinsic Rate of Increase, *r* (d^−1^)	Finite Rate of Increase, *λ* (d^−1^)	Net Reproductive Rate, *R*_0_ (Offspring)	Mean Generation Time, *T* (d)
H_2_O	0.2427 ± 0.0090 ab	1.2746 ± 0.0115 ab	70.4682 ± 10.1416 ab	17.5360 ± 0.1570 b
8010	0.2529 ± 0.0107 a	1.2878 ± 0.0137 a	69.7647 ± 10.9253 ab	16.7840 ± 0.2340 c
8010AKi	0.2510 ± 0.0089 a	1.2852 ± 0.0114 a	81.9904 ± 11.3744 a	17.5590 ± 0.2170 b
8010:8010AKi of 1:9	0.2065 ± 0.0115 c	1.2293 ± 0.0141 c	45.9127 ± 8.9724 bc	18.5360 ± 0.2660 a
8010:8010AKi of 3:7	0.2153 ± 0.0117 bc	1.2403 ± 0.0145 bc	52.2844 ± 10.1531 abc	18.3760 ± 0.2280 a
8010:8010AKi of 5:5	0.2383 ± 0.0115 abc	1.2691 ± 0.0146 abc	66.1250 ± 12.3838 abc	17.5920 ± 0.2250 b
8010:8010AKi of 7:3	0.2145 ± 0.0099 c	1.2393 ± 0.0122 c	56.4240 ± 9.3667 abc	18.7990 ± 0.2530 a
8010:8010AKi of 9:1	0.2097 ± 0.0147 c	1.2333 ± 0.0180 c	39.8571 ± 9.3817 c	17.5760 ± 0.2460 b

Different letters following mean ± SE in the same row represent significant differences among species based on the paired bootstrap test (200,000 bootstraps, *p* < 0.05).

## Data Availability

Data are available from the article and from authors on request.

## Supplementary Materials

The following supporting information can be downloaded at: https://www.mdpi.com/article/10.3390/ijms23010444/s1.
