# Supplementary material for: The Combination of Bacillus Thuringiensis and Its Engineered Strain Expressing dsRNA Increases the Toxicity against Plutella Xylostella
Source: Int J Mol Sci. 2021 Dec 31;23(1):444. doi: 10.3390/ijms23010444 (PMC8745139; doi:10.3390/ijms23010444)
Supplement: Supplementary file 1 [file ijms-23-00444-s001.zip › ijms-1501585-supplementary.pdf]

Supplementary information

Table S1 Life table statistics of *P. xylostella* populations in different treatments

Parameter		H_2O		8010		8010AKi		8010: 8010AKi of 1:9	
		n	Mean ± SE	*n*	Mean ± SE	*n*	Mean ± SE	*n*	Mean ± SE
Developmental duration/d	Egg	85	1.88 ± 0.04 a	73	1.45 ± 0.06 d	74	1.72 ± 0.05 b	70	1.56 ± 0.06 cd
	Larva	71	9.17 ± 0.07 c	56	8.89 ± 0.10 d	62	9.76 ± 0.10 ab	56	9.98 ± 0.12 a
	Pupa	71	3.94 ± 0.06 ab	56	3.84 ± 0.05 ab	62	3.85 ± 0.06 ab	53	3.64 ± 0.09 c
	Preadult	71	14.97 ± 0.10 c	56	14.12 ± 0.13 d	62	15.29 ± 0.13 ab	53	15.09 ± 0.13 bc
	Adult	71	18.93 ± 0.75 a	56	17.95 ± 0.89 a	62	18.35 ± 0.92 a	53	18.30 ± 0.98 a
	Adult (female)	37	16.27 ± 0.75a bc	32	17.06 ± 1.05 ab	39	14.82 ± 0.76 bcd	24	14.75 ± 1.02 abcd
	Adult (male)	34	21.82 ± 1.16 ab	24	19.12 ± 1.54 b	23	24.35 ± 1.46 a	29	21.24 ± 1.36 ab
Longevity/d		119	21.97 ± 1.43 a	102	19.34 ± 1.49 abc	104	21.33 ± 1.58 a	126	16.18 ±1.39 cd
Longevity (female)/d		37	31.19 ± 0.73 ab	32	31.25 ± 1.08 ab	39	30.05 ± 0.79 abc	24	29.75 ± 1.09 abc
Longevity (male)/d		34	37.20 ± 1.16 abc	24	33.17 ± 1.50 c	23	39.74 ± 1.54 a	29	36.41 ± 1.37 abc
Sex ratio (% female)		37	31.09 ± 0.04 ab	32	31.37 ± 0.05 ab	39	37.50 ± 0.05 a	24	19.05 ± 0.04 cd
Larval mortality		71	0.12 ± 0.03 ab	56	0.17 ± 0.04 ab	62	0.12 ± 0.03 ab	56	0.11 ± 0.03 ab
Preadult survival rate		71	0.60 ± 0.04 a	56	0.55 ± 0.05 abc	62	0.60 ± 0.05 ab	53	0.42 ± 0.04 cd
Fecundity/egg		37	209.50 ± 11.09 a	32	222.38 ± 12.49 a	39	218.64 ± 12.63 a	24	241.04 ± 16.12 a
Adult pre-oviposition period/d		37	0.11 ± 0.08 b	32	0.31 ± 0.18 b	39	0.00 ± 0.00 b	23	1.09 ± 0.09 a
Total preoviposition period/d		37	15.03 ± 0.14 c	32	14.50 ± 0.23 d	39	15.23 ± 0.15 c	23	16.09 ± 0.23 a
Oviposition period/d		37	11.84 ± 0.62 a	32	10.88 ± 0.78 ab	39	10.44 ± 0.60 ab	23	11.35 ± 0.69 a

Parameter		8010: 8010AKi of 3:7		8010: 8010AKi of 5:5		8010: 8010AKi of 7:3		8010: 8010AKi of 9:1	
		n	Mean ± SE	*n*	Mean ± SE	*n*	Mean ± SE	*n*	Mean ± SE
Developmental duration /d	Egg	71	1.68 ± 0.06 bc	57	1.75 ± 0.06 ab	77	1.60 ± 0.06 bcd	62	1.61 ± 0.06 bcd
	Larva	51	9.47 ± 0.16 bc	48	9.79 ± 0.16 ab	65	10.02 ± 0.16 a	41	9.83 ± 0.24 ab
	Pupa	51	3.69 ± 0.12 bc	47	3.91 ± 0.07 ab	63	3.98 ± 0.09 a	40	3.83 ± 0.07 abc
	Preadult	51	14.84 ± 0.17 c	47	15.47 ± 0.17 ab	63	15.54 ± 0.17 a	40	15.15 ± 0.20 abc
	Adult	51	17.33 ± 0.93 a	47	17.32 ± 1.08 a	63	19.00 ± 0.84 a	40	18.50 ± 1.04 a
	Adult (female)	24	14.21 ± 1.00 cd	24	12.71 ± 0.96 d	31	17.29 ± 0.99 a	17	14.06 ± 0.86 cd
	Adult (male)	27	20.11 ± 1.32 b	23	22.13 ± 1.40 ab	32	20.66 ± 1.29 ab	23	21.78 ± 1.35 ab
Longevity/d		109	16.94 ± 1.44 bcd	80	20.79 ± 1.75 ab	125	19.45 ± 1.46 abc	105	15.13 ± 1.50 d
Longevity (female)/d		24	29.21 ± 1.00 bc	24	27.88 ± 0.99 c	31	32.52 ± 1.00 a	17	29.29 ± 0.87 bc
Longevity (male)/d		27	34.81 ± 1.36 bc	23	37.91 ± 1.43 ab	32	36.50 ± 1.37 abc	23	36.87 ± 1.37 abc
Sex ratio (% female)		24	22.02 ± 0.04 bcd	24	30.00 ± 0.05 abc	31	24.80 ± 0.04 bcd	17	16.19 ± 0.04 d
Larval mortality		51	0.18 ± 0.04 ab	48	0.11 ± 0.04 ab	65	0.10 ± 0.03 b	41	0.20 ± 0.04 a
Preadult survival rate		51	0.47 ± 0.05 bcd	48	0.59 ± 0.06 ab	63	0.50 ± 0.04 abcd	40	0.38 ± 0.05 d
Fecundity/egg		24	237.46 ± 17.21 a	24	220.42 ±17.17 a	31	227.52 ± 13.26 a	17	246.18 ±19.43 a
Adult pre-oviposition period/d		23	1.00 ± 0.00 a	24	0.08 ± 0.08 b	31	1.06 ± 0.06 a	16	0.00 ± 0.00 b
Total preoviposition period/d		23	15.96 ± 0.30 ab	24	15.25 ± 0.20 bc	31	16.29 ± 0.23 a	16	15.25 ± 0.27 bc
Oviposition period/d		23	9.96 ± 0.74 ab	24	9.13 ± 0.79 b	31	10.97 ±0.54 ab	16	10.38 ± 0.84 ab

Different letters following mean ± SE in the same row indicate significant differences among all the treatment species using the paired bootstrap test (200000 bootstraps, $P < 0.05$).

Table S2 Sequences of primers used in the experiments

Name	Sequence (5’-3’)	Product size / bp	Restriction enzyme
01F	GTCTGGATCCGAAATTAGTTATACAAGCATT	1072	*Bam*HI
01R	ACATGCATGCTTTTCTTCCTCCCTTTCTT		*Sph*I
02F	TGCACTGCAGGAAATTAGTTATACAAGCATT	1072	*Pst*I
02R	AGATGTCGACTTTTCTTCCTCCCTTTCTT		*Sal*I
03F	ACATGCATGCCCGCCAACGCTTGCCGCTT	325	*Sph*I
03R	TGGTACTTGGACGCCACCT		-
F	ATCGTGGATACAATCGTCGAC	1115	
Rm	CGCCAAGCTTGTATGCCTGCAGG		
Fm	CCTGCAGGCATACAAGCTTGGCG	382	
R	GCTCACATGTTCTTTCCTGC		
Bt-F	TAAGAAAGGGAGGAAGAAAAGTCG	396	-
Bt-R	TGATAAGAAAGGGAGGAAGAAAAGC		-
AKiF	CTCCTCCAGCTTGGTCTTGTC	304	
AKiR	CAACGCTTGCCGCTTCTG		
Cry1F	ATCACTGTCGCTTCGCATGTTTGACTTTCTC	238	-
Cry1R	TCGAATTGAATTTGTTCCGGCAGAAGTA		-
AKF	AGAACTGGGGTGATGTTGAGAC	150	-
AKR	CCTTCTCCTCCATCTCCTTGTA		-
RF	CGGTCGTGCCTACCACAAATACA	89	-
RR	CGTGAGGATGCTCCACAGGGT		-

“__” stands for restriction enzyme and “__” for base mutation sequence.

Additional methodology

Since in the plasmid pHT305AKR, AKi harbors one *Sph*I restriction site at its one end and pHT304 harbors one *Sph*I restriction site in its sequence, we conducted SOE-PCR (Slicing by Overlapping Extension Polymerase Chain Reaction) to delete the latter. Primers F/Rm and R/Fm (Table S1) were used to amplify two fragments, respectively. For F/Rm, the reaction procedure was similar with the pro3α(+) amplification except for the first annealing temperature time up to 65°C and reducing by 1 °C per cycle; for R/Fm, it had only 25 repeats of 98 °C for 10 s, 60°C for 15 s and 72 °C for 30 s and no change for initial degeneration and final extension. Mixed those products at ratio of 1:3 as the template to conduct SOE-PCR using primers F/R. Reaction procedure was 98 °C for 3 min, followed by 20 cycles of 98 °C for 10 s, 55 °C for 60 s (reduced by 0.5 °C per cycle) and 72 °C for 5min, then paused for 10 min to add primers F/R and 0.15 ul enzyme, and then restarted it using following steps: 98 °C for 3 min, 15 cycles of 98 °C for 10 s, 65 °C for 20 s (reduced by 0.8 °C per cycle), 72 °C for 2 min, followed by 20 cycles of 98 °C for 10 s, 53 °C for 20 s 72 °C for 2 min, and 72 °C for 5 min. At last, the products got by SOE-PCR and the plasmid pHT305AKR were both double-digested by *Sal*I (TaKaRa, Japan) and *Pci*I (TaKaRa, Japan), then constructed to form the final plasmid pHT305AKR2 with the help of T4 ligase (TaKaRa, Japan).

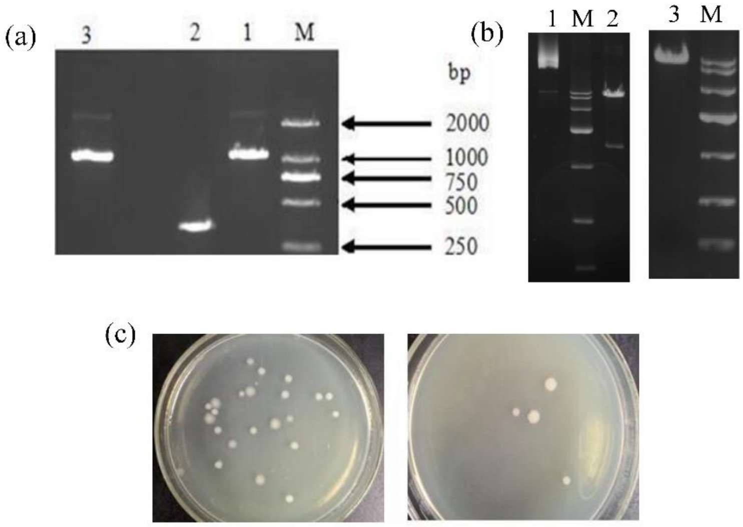

Figure S1. Construction of engineered Bt expressing *PxAK* dsRNA. (a) PCR amplification of AKi and promoter sequence: ‘M’ stands for DNA marker 2000, 1 for pro3α(+), 2 for AKi and 3 for pro3α(-). (b) Identification of plasmid by restriction enzyme digestion. M, DNA marker 10000; 1, pHT305AKR plasmid; 2, digestion products of pHT305AKR plasmid by *Sph*I; 3, digestion products of pHT305AKR2 plasmid by *Sph*I and . (c) Colonies of transformed Bt strains: the left one is BMB171AKi and the right one is 8010AKi.

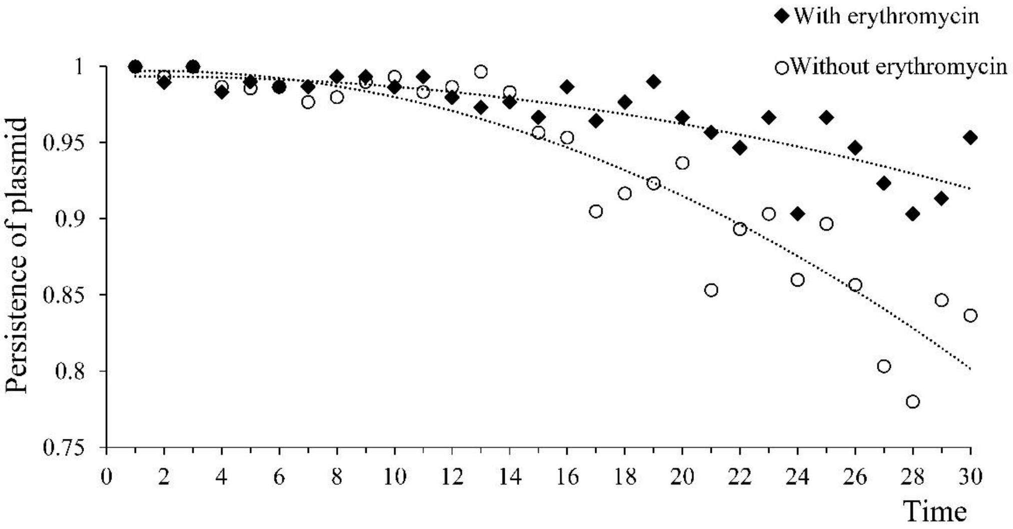

Figure S2. Effects of erythromycin on the plasmid stability in 8010AKi in LB medium. The stability plasmid of pHT305AKR2 was detected and calculated by the method described by Harwood and Cutting (1991). The culture was examined every six hours for 30 times. The dot lines represent the trends of persistence of plasmid.

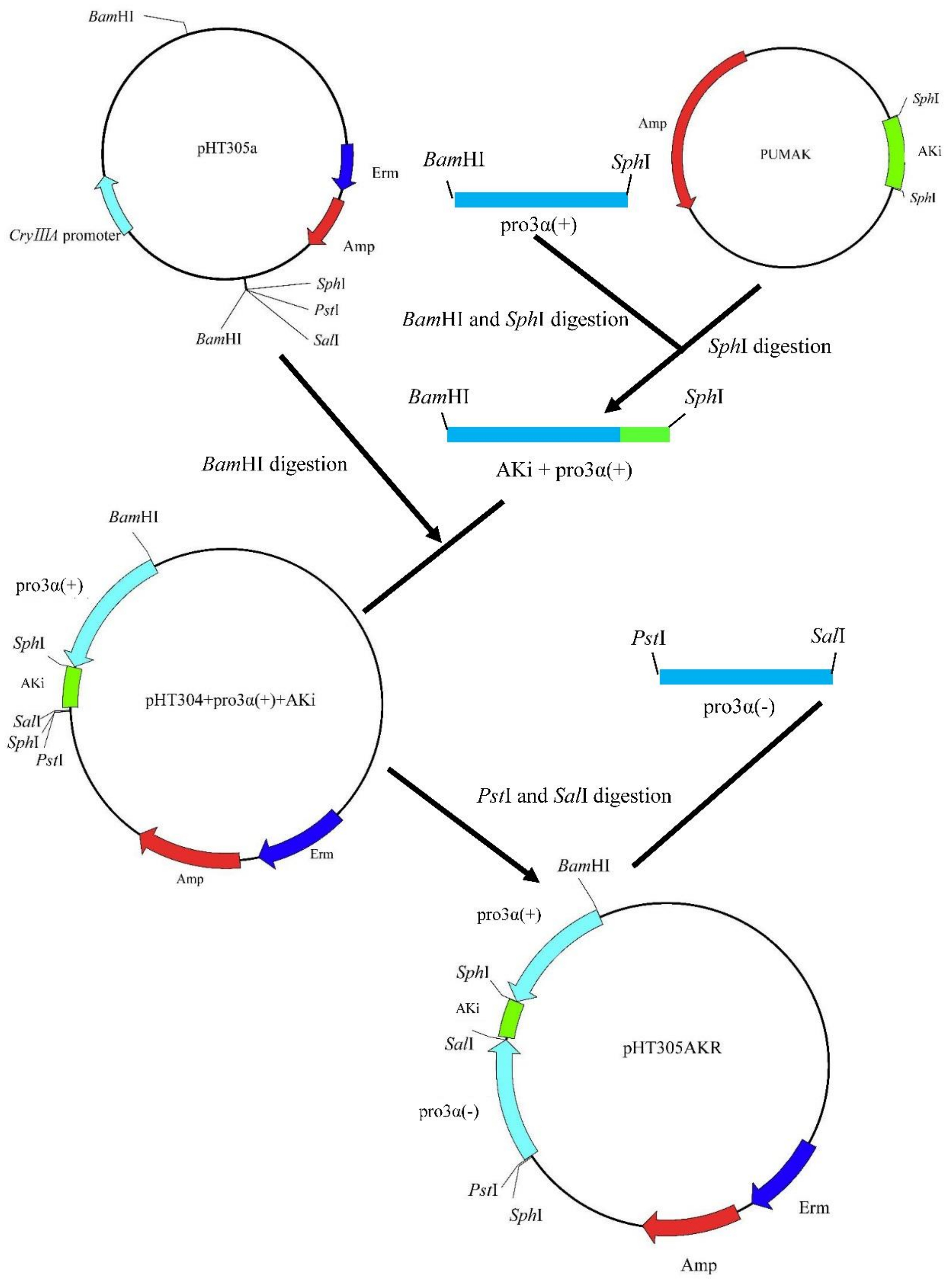

Figure S3. Construction of dsRNA expression vector of *PxAK*. A fragment of 325-bp *PxAK* (AKi) was chosen to be the target of RNAi and cloned from the cDNA of *P. xylostella*, and then inserted into plasmid PUM-T (BioTeke, China) to gain plasmid PUMAK. The plasmid PUMAK was digested by *Sph*I (TaKaRa, Japan) to obtain the

fragment of AKi. AKi was linked to pro3α(+) to acquire the fragment of AKi+pro3α(+). The plasmid pHT305a was digested by *Bam*HI (TaKaRa, Japan) to obtain the fragment pHT304. Then the fragment AKi+pro3α(+) was linked to the fragment pHT304 to obtain the intermediate cloning vector pHT304+pro3α(+)+AKi. At last the pro3α(-) was linked to the intermediate vector to get the plasmid pHT305AKR containing pHT304+pro3α(+)+AKi+pro3α(-).
